# Clinical Efficacy, Pharmacokinetics, and Safety of the Available Medical Options in the Treatment of Endometriosis-Related Pelvic Pain: A Scoping Review

**DOI:** 10.3390/ph16091315

**Published:** 2023-09-18

**Authors:** Mislav Mikuš, Marina Šprem Goldštajn, Antonio Simone Laganà, Franka Vukorepa, Mario Ćorić

**Affiliations:** 1 Department of Obstetrics and Gynecology, Clinical Hospital Center Zagreb, 10 000 Zagreb, Croatia; marina.goldstajn@gmail.com (M.Š.G.); franka.vukorepa@gmail.com (F.V.); mcoric77@gmail.com (M.Ć.); 2 Unit of Obstetrics and Gynecology, “Paolo Giaccone” Hospital, Department of Health Promotion, Mother and Child Care, Internal Medicine and Medical Specialties (PROMISE), University of Palermo, 90127 Palermo, Italy; antoniosimone.lagana@unipa.it

**Keywords:** endometriosis, pelvic pain, pharmacotherapy, pharmacokinetics, non-steroidal anti-inflammatory drugs, progestins

## Abstract

Background: In this scoping review, we sought to identify published studies evaluating the drugs currently used in the treatment of endometriosis-related pelvic pain, with reflection on their chemical properties, pharmacokinetics, safety profile, and clinical efficacy. Methods: A literature search was conducted with the use of the PubMed and EMBASE electronic databases, focusing on identifying articles published in English between January 1990 and 2023. Results: Based on the included studies, current therapy options for the treatment of endometriosis-related pain identified and reviewed in this article were: (1) non-steroidal anti-inflammatory drugs; (2) combined oral contraceptive (COCs); (3) progestins; (4) gonadotropin-releasing hormone agonists and antagonists; (5) aromatase inhibitors (AIs); (6) selective estrogen and progesterone receptor modulators; and (7) levonorgestrel-intrauterine device. Conclusions: Based on the published evidence, clinicians should consider NSAIDs, COCs, and progestins as the first-line medical therapies. Compared with second-line options, such as GnRH agonists/antagonists or AIs, the abovementioned first-line options are well tolerated, efficacious, and exhibit lower overall price. Future research priorities should be to identify novel target therapies and to evaluate the effects of available drugs through different routes of administration.

## 1. Introduction

Endometriosis is one of the most common chronic gynecological conditions associated with cyclic pelvic pain, subfertility, or both [1]. It affects approximately 20% of women hospitalized for pelvic pain and 50% of infertile women [2,3]. The symptoms of endometriosis vary from mild to severe, but in most cases, women suffering from endometriosis need an important monthly intake of analgesics and generally have a poor health-related quality of life [4,5]. Furthermore, frequent misdiagnosis leads to prolonged delivery of effective pharmacotherapy and causes a substantial economic burden [1]. 

During the past decade, several medical and surgical treatment options were adopted and demonstrated various successes, depending on the disease stage [6]. Although the surgical approach is indicated for temporary pain relief in patients seeking spontaneous conception, the effect of surgery on the pain component is not always successful [4]. Moreover, ovarian surgery can deteriorate overall gonadal function and ovarian reserve and is also associated with other surgical complications, depending on the site of the endometriotic lesions [7]. Although adequate quality evidence is lacking, a recent meta-analysis demonstrated no benefit of operative laparoscopy with respect to fertility-related or adverse outcomes when compared to diagnostic laparoscopy [8]. Given the published evidence, the recurrence rate of pain symptoms after surgery is not negligible [9]. Another aspect of surgical management is patients’ preference, which has yet to be determined in prospective trials [8]. 

Current medical options in endometriosis treatment are designed for women who desire to delay or avoid surgery, favoring a long-term solution to control their symptoms, such as chronic pelvic pain [10]. Long-term pharmacotherapy ideally should balance clinical efficacy with an acceptable safety profile. However, the aim of endometriosis pharmacotherapy is to ameliorate chronic pelvic pain and to preserve a woman’s desire to conceive.

In this scoping review, we sought to identify the published studies evaluating the drugs currently used in the treatment of endometriosis-related pelvic pain, with particular reflection on their chemical properties, pharmacokinetics, safety profile, and clinical efficacy.

## 2. Results

Based on included studies, current therapy options for the treatment of endometriosis-related pain identified and reviewed in this article (summarized in Table 1) were: (1) non-steroidal anti-inflammatory drugs (NSAIDs); (2) combined oral contraceptive (COCs); (3) progestins; (4) gonadotropin-releasing hormone agonists (GnRH agonists); (5) gonadotropin-releasing hormone antagonists (GnRH antagonists); (6) aromatase inhibitors (Ais); (7) selective estrogen receptor modulators (SERMs); (8) selective progesterone receptor modulators (SPRMs), and (9) levonorgestrel-intrauterine device (LNG-IUD).

## 3. Discussion

### 3.1. Non-Steroidal Anti-Inflammatory Drugs (NSAIDs)

Since their introduction, NSAIDs have been among the most widely used over-the-counter drugs across the world, mostly used for the treatment of patients suffering from pain and inflammatory conditions [10]. 

According to their chemical characteristics, NSAIDs can be categorized based on their selectivity for inhibiting cyclooxygenase/prostaglandin-endoperoxide synthase (PGHS) enzymes. Another classification, although rarely used in clinical practice, implicates their chemical structure, categorizing it as major derivatives of salicylic acid, acetic acid, enolic acid, anthranilic acid, or propionic acid [11]. Furthermore, NSAIDs can be divided according to the plasma half-life (t½) into short-acting (t½ < 6 h), such as aspirin and ibuprofen, and long-acting (t½ > 10 h), such as naproxen. 

One of the NSAIDs’ main advantages is excellent bioavailability regardless of the administration route and moderate to high lipid solubility, which allows their penetration of the blood–brain barrier [12]. A high predilection to the plasma proteins (i.e., albumin) influences drug elimination; hepatic metabolism, mainly by the microsomal enzymes cytochrome P450 (CYP), clears out the NSAIDs as inactive metabolites that are excreted in urine and bile. Among the group, CYP2C9 predominantly contributes to NSAID clearance, while allelic variations in this protein affect the pharmacotherapeutic efficacy and are subject to pharmacogenomic variability in population studies [13,14]. 

From a clinical standpoint, although widely used to treat endometriosis-related pain symptoms, current evidence to support the use of NSAIDs is of very low-quality owing to the risk of bias and imprecision [15]. Interestingly, there have been no conducted high-quality studies in the last three decades [15]. A study from Kauppila and Ronnberg demonstrated better clinical effects of naproxen sodium compared with a placebo for the management of pain caused by endometriosis [16]. There is no comparing evidence in terms of which NSAID is more effective. Because of the well-known atherothrombotic vascular events related to PGHS-2 inhibitors, non-specific inhibitors of both enzymes present the only reasonable option for controlling pain symptoms in this drug group [17]. Furthermore, clinicians must be aware that habitual use of NSAIDs is also associated with nephrotoxicity and potential renal failure [18]. 

### 3.2. Combined Oral Contraceptives (COCs)

Combined oral contraceptives (COCs) are a long-term safety option for treating endometriosis-related pain [10]. COCs can be used in oral formulations, vaginal rings, or transdermal patches, either in sequential or continuous regimes, using low or high dosages of particular compounds. Although COCs offer several clinical advantages, such as contraception, regulation of the menstrual cycle, low rates of adverse events, and low price, their pharmacokinetic profile exhibit large intersubject variability, mediated by CYP enzyme activity and ethnic differences [19]. Nowadays, an increasing magnitude of obesity in the population certainly takes the majority of the intersubject variability; however, isolated obesity effects on COCs pharmacokinetics are not clearly understood. Several pharmacokinetical studies concluded that altered COC metabolism in obese patients is a common event [20]. Briefly, obesity influences each of the four primary processes involved in the passage of a drug through the body: absorption, distribution, metabolism, and excretion. One of the key determinants in COCs’ clinical effect is hepatic, first-pass metabolism, which defines COCs bioavailability. In terms of estrogen components, bioavailability for ethinyl estradiol (which is the most used estrogen component) shows a broad range, from 25% to 65% [21]. For most synthetic progestins, bioavailability is up to 90% [20]. In normal-weight women, the time to maximum concentration in the systemic circulation is 1–3 h, and plasma half-life (t½) ranges from 12 to 24 h, depending on the formulation [19]. Another concern was growing evidence about the possible interactions between COCs and various therapeutic agents [22,23]. 

COCs have been used for many years as the first-line therapy, exhibiting many beneficial effects on endometriosis-related symptoms by the induction of the atrophy of the eutopic and ectopic endometrium, limiting retrograde menstruation, inhibition of ovulation and anti-inflammatory and proapoptotic effects on endometriotic foci [24]. In terms of clinical effectiveness (resulting in lowered endometriosis-related pain), almost one-third of patients treated do not have an adequate response, which may be in part due to progesterone resistance [10]. However, several randomized controlled trials have shown efficacy with COCs in treating pain associated with endometriosis [25,26,27,28]. Regarding the other formulations (vaginal ring and/or transdermal patch), conclusions can be drawn from patient preference prospective cohort studies [29,30]. A total of 143 women with deep infiltrating endometriosis were treated either with a desogestrel-only contraceptive pill or with the sequential combined contraceptive vaginal ring for 12 months. The final results clearly demonstrated clinical efficacy in both study arms, with higher patient satisfaction in the desogestrel-only arm group [29]. 

### 3.3. Progestins

Progestins represent a great clinical alternative in women with endometriosis-related pain, and their multiple administration routes can provide better treatment adherence compared to COCs [31]. Regarding clinical efficacy, progestins are effective in alleviating pain in women with gastrointestinal and rectovaginal endometriosis and have shown results comparable to surgery in the treatment of endometriosis-related dyspareunia [32]. The main pharmacological effects of progestins can be summarized as altering the frequency and intensity of pulsatile gonadotropin-releasing hormone (GnRH), therefore acting on the secretion of follicle stimulating and luteinizing hormone and, consequently, causing an ovarian steroidogenesis suppression after continuous administration. A mechanism that is relevant to endometriosis-related pain is causing the decidualization of ectopic and eutopic endometrium, respectively. According to their chemical structure, progestins can be classified as 17-hydroxyprogesterone and 19-nortestosterone derivatives, administered via several different routes such as oral formulations, depot subcutaneous and intramuscular injections, transdermal patches, vaginal gels or rings and intrauterine devices [31,33]. The concentration of the progestin available to exhibit biological actions in target tissues is influenced by factors such as route of administration, metabolism, and bioavailability. Like in orally administered COCs, progestins taken orally undergo hepatic, first-pass metabolism, resulting in a significant reduction of active compound plasma concentration compared to parenterally administered progestins [33]. Parenterally administered progestins are also significantly metabolized in the liver; however, experimental studies suggest that metabolism may also occur at the site of administration or the target sites expressing steroid-metabolizing enzymes, such as skin, vagina, endometrium, and uterus [34,35]. The bioavailability of progestins is similar to that of COCs; however, current data is limited and is influenced by the high inter-individual variability [33]. In terms of plasma half-life, the relatively short plasma half-life of progestin explains the necessity of daily oral use in order to maintain clinical efficacy. Although there are many progestins existing on the market, complete insight into pharmacokinetics is limited, and further investigations are warranted.

According to the latest Cochrane review, progestins are particularly efficacious in women suffering from dysmenorrhea, offering good long-term safety and tolerability [36]. However, only one study (out of 13 included) using 100 mg daily dose of medroxyprogesterone acetate (MPA) was found to be effective compared to placebo in controlling endometriosis-related pain [36]. Several published randomized, controlled studies afterward support the employment of progestin monotherapy in alleviating pelvic pain associated with endometriosis [37]. Moreover, progestin clinical use receives encouragement in controlling pain symptoms in patients with deep infiltrating endometriosis [38,39]. Dienogest, a fourth-generation selective progestin with a direct anti-inflammatory effect, received justified attention in controlling endometriosis-related pain and disease recurrence [31]. A systematic review by Andres and colleagues showed excellent clinical efficacy of dienogest in treating endometriosis, equivalent to GnRH analogs, with better tolerability and safety profile [40]. Another benefit of progestin use compared to COCs is less thrombotic risk and better tolerability (i.e., progestins can be administered to patients suffering from migraine with aura in patients younger than 35 years old) [41,42]. Although well tolerated, cost-effective, and with limited adverse events, progestins do not eradicate disease, and symptom recurrence is frequent after treatment discontinuation [42]. 

### 3.4. Gonadotropin-Releasing Hormone Agonists

Since the discovery of gonadotropin-releasing hormone (GnRH, also called luteinizing hormone-releasing hormone, LHRH), several GnRH analogs have been developed for treating women with various gynecological conditions, such as uterine fibroids, endometriosis, and central precocious puberty [43]. GnRH is a small decapeptide (pyroGlu-His-Trp-Ser-Tyr-Gly-Leu-Arg-Pro-Gly-amide), synthesized and stored in the medial basal hypothalamus and has a pivotal role in regulating reproduction by regulating the secretion of gonadotropins by the pituitary. The action of GnRH and its analogs are mediated by G-protein coupled receptors, which are expressed on the membranes of the pituitary gonadotrophs, placenta, uterus, ovary, breast, and prostate gland [44,45,46]. 

Among the GnRH agonists, leuprolide acetate is not only the most used but also yields quite robust evidence about its mechanisms of action, clinical pharmacology, safety, and tolerability [47]. Leuprolide acetate is a synthetic nonapeptide GnRH analog, with the chemical name 5-oxo-L-prolyl-L-histidyl-L-tryptophyl-L-seryl-L-tyrosyl-D-leucyl-L-leucyl-L-arginyl-N-ethyl-L-prolinamide acetate, available in several different administration routes and doses [48]. Compared with endogenous GnRH, leuprolide acetate is more potent due to its longer half-life and increased affinity for GnRH receptors, with similar bioavailability regardless of administration route (subcutaneous vs. intravenous) [49,50,51]. Pharmacokinetical aspects of leuprolide acetate after subcutaneous and intramuscular administration are summarized in Table 1. GnRH agonists have also been developed for nasal administration (i.e., nafarelin).

Although considered an agonist, when administered in therapeutic doses continuously, it produces an inhibition of the hypophyseal–gonadal axis within 2–4 weeks [48]. However, these actions are entirely reversible on discontinuation of treatment. Although the main mechanism of action can be modified with treatment discontinuation, adverse events such as altered lipid profile, depression, hot flushes, and loss of bone mineral density limit their long-term use [48]. An extensive systematic review and meta-analysis published 12 years ago investigated the use of GnRH agonists at different doses, regimens, and routes of administration for improving endometriosis-related pain symptoms [52]. There were 41 randomized controlled trials (RCTs) included in the final analysis with a total of 4935 women. Brown et al. found GnRH agonists as a better alternative than placebo and danazol in relieving endometriosis-related pain, especially dysmenorrhea [52]. Several RCTs have demonstrated great clinical efficacy of GnRH agonists compared to placebo or no treatment [53,54,55,56]; however, there is limited evidence in terms of optimal dosage, duration of therapy, and route of administration with GnRH agonists. In the abovementioned studies, GnRH agonists were used in subcutaneous, intramuscular, and intranasal formulations in different daily and monthly regimens. GnRH agonists have been compared to almost every of the currently available hormonal treatments used for treating endometriosis-related pain. In brief, compared to progestins and COCs, GnRH agonists decrease pain symptoms and improve quality of life, however, without inter-group differences [57,58]. Regarding the different routes of administration between GnRH agonists, current evidence shows that there was no statistically significant difference between the groups for pelvic pain, deep dyspareunia, and dysmenorrhea [59,60,61]. Furthermore, GnRH agonists can be used in the postoperative period in the prevention of endometriosis recurrence. Zheng et al. concluded in their meta-analysis that longer-term (6 months) postoperative administration of GnRH agonists can decrease the recurrence risk of endometriosis [62]. 

### 3.5. Gonadotropin-Releasing Hormone Antagonists

**Figure 1:**
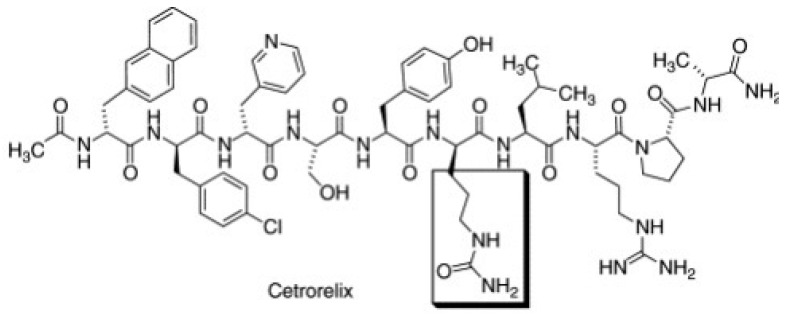
The biochemical structure of GnRh antagonist cetrorelix.

Unlike the GnRH agonists, GnRH antagonists do not induce an initial gonadotropin release flare but cause an immediate and reversible suppression of gonadotropin secretion by competing with the endogenous GnRH for its pituitary receptors. Another advantage, compared with GnRH agonists, is the dose-dependent effect: in particular, suppression of pituitary and ovarian hormones is maintained partially at lower doses, while higher doses are associated with full suppression [63]. Figure 1 clarifies the biochemical structure of GnRH antagonists and their crucial positions, such as positions 2 and 3, which are responsible for gonadotropin release, and position 6 involved in enzymatic cleavage. In terms of pharmacokinetic properties, with special regard to maximum antagonist administration (Cmax), a linear pattern occurs following single or multiple doses [63,64]. 

In 12 premenopausal female volunteers, relugolix oral administration of a single dose (40 mg) resulted in a Cmax of 29.05 ng/mL after 90 min (t_1/2_ was 45.42 h) [65]. The clearance of GnRH antagonists is predominantly metabolic and hepatic [66]. The last generations of GnRH antagonists are characterized by low histamine-releasing potency, which improves their overall safety and tolerability [63]. 

Because of their pharmacodynamics, they can be started when they are needed during controlled ovarian hyperstimulation (COH) to prevent luteinizing hormone (LH) surge [66]. Similar to the GnRH agonists, GnRH antagonists can be translated into everyday clinical practice for the treatment of endometriosis, uterine fibroids, and benign prostatic hyperplasia [10,65]. Regarding administration routes, GnRH antagonists are available as oral nonpeptide forms and as an injectable formulation. One of the very recent randomized controlled trials studied the effect of a new investigational oral GnRH antagonist, linzagolix, on endometriosis-associated pain [67]. Compared with placebo, doses ≥ 75 mg resulted in a significantly greater proportion of responders for overall pelvic pain, dysmenorrhea, and non-menstrual pelvic pain at 12 weeks. In addition, mean bone mineral density (BMD) loss at 24 weeks increased in a dose-dependent fashion for a 200 mg regimen [67]. A Phase 2 randomized controlled study evaluated the safety and efficacy of elagolix for treating endometriosis-related pain compared to placebo on a total of 155 women with laparoscopically confirmed endometriosis [68]. In this study, elagolix demonstrated a great clinical effect on reducing dysmenorrhea and nonmenstrual pelvic pain scores with acceptable safety and tolerability. Two double-blind, randomized, 6-month phase III trials (Elaris EM-I and Elaris EM-II) evaluated the effects of 150 mg once daily and 200 mg twice daily regimens compared with placebo in women with laparoscopically diagnosed endometriosis and moderate to severe pain symptoms [69]. Although some proportion of hypoestrogenic adverse events were reported in elagolix study groups, both higher and lower doses of elagolix were effective in improving dysmenorrhea and nonmenstrual pelvic pain during a 6-month period in women with endometriosis-associated pain. Moreover, both Elaris trials indicate that elagolix produces improvements in all aspects of health-related quality of life that are clinically meaningful to patients [69]. From the economic burden standpoint, a cost-effectiveness analysis of elagolix versus leuprolide acetate found that elagolix was dominant over leuprolide acetate in the treatment of moderate to severe endometriosis-related pain over one- and two-year-time span [69]. More recently, SPIRIT trials have demonstrated that once-daily relugolix combination therapy significantly improved endometriosis-associated pain and was well tolerated among 638 enrolled patients [70]. According to the SPIRIT trials, this oral therapy has the potential to address the unmet clinical need for long-term medical treatment for endometriosis, reducing the need for opioid use or repeated surgical treatment [70].

### 3.6. Aromatase Inhibitors

The rationale for the clinical use of third-generation aromatase inhibitors (AIs) is a selective inhibition of enzymatic aromatase activity in peripheral tissues, resulting in suppression of estrogen production. Their clinical use is mostly confined to adjuvant and neoadjuvant settings in breast cancer treatment; however, AIs have shown size-reducing effects on endometriotic lesions [71]. Third-generation AIs are more selective and pharmacologically more potent than the previous two generations of AIs, also exhibiting a favorable pharmacokinetic profile, allowing once-daily administration [72]. To be more precise, letrozole (mostly used in prospective studies on endometriosis-related pain) is characterized by rapid absorption, a long half-life, low plasma clearance, and a high-volume distribution [72,73]. Metabolism and excretion are hepatic and metabolic, mediated by CYP3A4 and CYP2A6 [73].

In regard to clinical efficacy, prospective studies on a limited number of patients have demonstrated the beneficial effect of AIs in improving endometriosis-related pain symptoms [74,75,76,77]. Furthermore, in postoperative surveillance, a combination of AIs with GnRH agonists for 6 months showed better results than GnRH agonists monotherapy in increasing the pain-free interval [78]. Another benefit of AIs was demonstrated in improving urinary symptoms in women with bladder endometriosis, intestinal symptoms in women with colorectal endometriosis, and in volume decrease of endometrial ovarian cysts [10]. However, due to their significant long-term adverse events, such as the increased risk of osteoporosis and bone fractures, AIs are not widely implemented in the clinical management of disease progression [75]. Therefore, AIs appear to be most beneficial in patients with recurrent endometriosis who have not had success with more conventional treatment protocols such as gonadotropin-releasing agonists/antagonists or steroidal analogs [76]. Due to side effects associated with systemic administration, vaginal rings were introduced as an alternative using the advantages of local administration [71,79]. A randomized phase I trial of pharmacokinetics, pharmacodynamics, safety, and tolerability of anastrozole administered vaginally showed no other significant adverse events or formation of ovarian cysts [80]. However, further clinical studies in terms of the adoption of this approach in endometriosis treatment are mandatory.

### 3.7. Selective Estrogen Receptor Modulators

As endometriosis is considered an estrogen-dependent disease, induction of a hypoestrogenic condition, whether with lowering circulating estrogen levels or with an antagonistic effect on estrogen receptors, is one major active mechanism using established medical agents. That hypothesis suggests that selective estrogen receptor modulators (SERMs) could be an option in the treatment of pelvic pain due to endometriotic lesions. SERMs bind to estrogen receptors (ER-α and ER-β) in target cells, acting as both ER agonist antagonists in different tissues, which makes them unique. Therefore, they have been proposed for the treatment of endometriosis and are under investigation. For instance, both raloxifene and tamoxifen exhibit ER agonist activity in bone and antagonist activity in the breast, but only tamoxifen manifests uterine agonist activity [81]. These compounds are well, and the most common adverse effects experienced in patients undergoing SERM therapy include vasomotor symptoms such as hot flashes and vaginal discharge. Interestingly, eventual fatal adverse effects like thromboembolic phenomenon, even though associated with this group of agents, are rare. Clinically used SERMs are administered orally and have great bioavailability, with the exception of raloxifene, which undergoes extensive first-pass metabolism to form glucuronide conjugates. They are all metabolized in the liver, eliminated in the bile, and excreted in the feces, with a small fraction eliminated by urine. Considering these elements, good liver function is important when considering SERMs for treatment. SERMs are very highly bound to plasma proteins (>95%), and terminal elimination half-lives range from 27.7 h to 7 days [82].

SERMs can be divided into four categories. First are the triphenylethylene derivatives like clomiphene and tamoxifen, used to treat ovarian anovulation and breast cancer, with their application limited due to endometrial stimulation; second are non-steroidal compounds like raloxifene, a benzothiophene derivative; third are indoles where bazedoxifene is the representative; and fourth are the steroidal compounds, for example, fulvestrant which is pure antiestrogenic agent [81]. Only a few substances from the SERM group have been investigated in endometriosis treatment.

As already mentioned, due to the estrogen-dependent nature of endometriosis, SERMs have been proposed for its treatment [83]. However, no SERMs have been reported to be effective in the treatment of endometriosis so far [84].

Tamoxifen is a first-generation SERM used for the treatment of breast cancer. Usage of tamoxifen as an alternative modality in the treatment of endometriosis was expected, especially for women desiring to conceive. However, after the global use of tamoxifen for breast cancer, occurrences of endometriosis were reported in post-menopausal patients who had been taking tamoxifen for the treatment of breast cancer [85]. As these effects of tamoxifen were considered to be derived from its estrogen receptor agonistic activity in the endometrium, other SERMs that have more selective estrogenic activity were evaluated.

Raloxifene has been used for the treatment of post-menopausal osteoporosis as it exerts an agonistic estrogen effect on bones and the cardiovascular system. However, in the mammary gland and uterus, it exhibits an estrogen-antagonist effect [86]. The rationale for medical therapy for women with endometriosis is the fact that raloxifene has a beneficial effect on bone density without concurrent endometrial stimulation. Tested in animal studies, it induced regression of endometriosis implants [87,88]. In a randomized clinical trial in biopsy-proven endometriotic lesions with chronic pelvic pain, the raloxifene group experienced significant pain comeback and had secondary surgery statistically significantly sooner than the placebo group. Patients with endometriosis-related pelvic pain following surgical treatment were randomly assigned to raloxifene or placebo for 6 months. The dose of raloxifene used was higher because of the available safety data, but the authors assumed that prolonged higher doses might have a stimulated effect on endometriotic lesions, which was not expected. The trial concluded that raloxifene statistically significantly shortened the time of return to chronic pelvic pain, and the study was halted prematurely because of that [89]. Bazedoxifene is a third-generation SERM with a good safety profile used primarily for post-menopausal osteoporosis prevention and treatment. In one animal study, it reduced the size of endometrial lesions with experimental evidence of an antiproliferative effect [90]. In addition, bazedoxifene was shown to decrease proliferating cell nuclear antigen and estrogen receptor expression in the endometrium of treated animals compared with controls. However, the effectiveness of bazedoxifene on endometriosis in humans has not been published.

SR-16234 is the most recent experimental SERM with antagonistic activity on ERα and partial agonistic activity on ERβ. The data from the clinical trial from 2018 suggests that SR-16234 may mitigate pelvic pain related to endometriosis at a 40 mg daily dosage by oral administration. As no other SERMs have shown such clinical efficacies in endometriosis, SR-16234 is the first SERM with reported efficacy in this field. The mechanism action of SR-16234 for endometriosis is not well clarified. Compared with 1st or 2nd generation SERMs, including tamoxifen or raloxifene, SR-16234 seems to be a purer ERα antagonist, and that may be one of the reasons it is effective for endometriosis-related pain. Its strong affinity to both ERα and ERβ might be important [57]. Further large-scale and placebo-compared clinical trials in the future are necessary for confirmation. The effort continues to find an “ideal SERM” as an alternative to other drugs for pain relief associated with endometriosis.

### 3.8. Selective Progesterone Receptor Modulators

Selective progesterone receptor modulators (SPRM) are a class of synthetic ligands that have a variable effect (agonist, antagonist, or mixed effect) on progesterone receptors from different targeted tissues. They have emerged as a possible treatment option for hormone-dependent conditions like uterine fibroids, endometriosis, breast cancer, and endometrial cancer. Due to its lesser effect on estradiol and androgen levels, SPRM treatment is not associated with systemic hypoestrogenic side effects [91]. Another advantage is that they can be administered orally or vaginally. They avoid the hypoestrogenic side effects of the alternative medical treatments because they maintain mid-follicular estrogen plasma levels. As a relatively huge proportion of benign gynecological conditions are hormonally influenced, SPRMs could show great treatment potential in the absence of the side-effect profile of the current pharmacological treatment options.

One of the potential theoretical pitfalls of SPRMs in everyday clinical practice could be a risk of unopposed endometrial estrogen exposure. Regarding endometrial morphology after SPRM use, some studies identified endometrial morphologic deviations due to the antiprogestin effect [92]. These endometrial morphologic deviations, so-called progesterone receptor modulator associated endometrial changes (PAEC), represent a spectrum of endometrial patterns that are histologically specified by cystically dilated glands, inactive epithelium, and few mitotic figures in a background of a compact non-decidualized stroma [93]. However, not only are these alterations reversible after the drug discontinuation, but they do not seem to contain any cytological atypia [94]. Although PAEC has been introduced as a benign endometrial alteration, further investigations are necessary in order to bold the overall safety of SPRMs [95].

The first SPRM discovered was mifepristone, which has both progesterone agonistic and antagonistic effects. In a phase II/III trial, the efficacy of mifepristone was observed in terms of symptom improvement, but adverse effects were noted in a significant number of patients. Administration of mifepristone (50 mg for 6 months) in patients with endometriosis has been reported to have a significant effect on symptoms [96]. A Cochrane systematic review of 10 randomized control trials involving 960 patients suggests that mifepristone improves endometriosis-associated dysmenorrhea and possibly dyspareunia. Amenorrhea and hot flashes have been reported as side effects, although the absence of menstruation may clearly be beneficial in women with endometriosis-associated heavy menstrual bleeding. However, no firm conclusions on dosing can be drawn based on the available data [97].

Asoprisnil is an 11β-benzaldoxime-substituted SPRM that has demonstrated mixed progesterone agonist/antagonist activity in both animal models and clinical trials in women [98]. In a randomized, placebo-controlled trial, asoprisnil (5, 10, or 25 mg) was administered for 12 weeks to women with a laparoscopic diagnosis of endometriosis who complained of moderate or severe pain. Significant reductions in nonmenstrual pelvic pain and dysmenorrhea were noted compared with placebo [99]. Clinical development was discontinued in 2007 due to the observation of abnormal endometrial changes in patients [100]. Like mifepristone, ulipristal acetate (UPA) was labeled an “antiprogestin” when it was developed, and only in recent years has it been classified as an SPRM [101]. It is a steroid drug currently approved for emergency contraception and as preoperative therapy for symptomatic women with uterine fibroids.

In 2018, the European Medicines Agency (EMA) warned of a serious risk of liver injury from UPA and recommended some measures to minimize adverse effects. The drug is contraindicated in patients with liver disease; liver tests are required before, during, and after administration of the drug; repeated therapies can only be offered to women who are not candidates for surgery; and patients must be fully informed of the risks. Further studies are needed to clarify the mechanisms of hepatotoxicity of UPA and to confirm that such prophylactic measures are indeed effective. However, the status of UPA as a drug responsible for drug-induced liver injury (DILI) has not been fully confirmed [102]. Current knowledge suggests that UPA may be responsible for idiosyncratic (rather than intrinsic) DILI, and monitoring liver health will help minimize the risks associated with its use [103]. Current EMA guidelines recommend the use of UPA only as the second-line treatment of uterine fibroids in premenopausal women for whom surgical procedures are not appropriate or have not worked [102,103].

A recent systematic review examined the endometrial effects of UPA ingestion in 10 studies of 1450 women. The review supports the current understanding of PAEC and shows that it is essentially a benign condition that is reversible after discontinuation of UPA use. However, most studies have limited follow-up and have used UPA in up to four intermittent courses, so further investigation is needed before it can be assumed that SPRMs, including UPA, are safe for long-term use [95]. To date, we have found no RCTs examining the role of UPA in the treatment of endometriosis and adenomyosis.

There is conflicting and limited evidence on the role of UPA in endometriosis. In animal models (rats with induced endometriosis), UPA was found to induce regression and atrophy of endometriosis lesions. This was associated with the upregulation of proapoptotic markers, decreased cell proliferation, and inflammatory markers [104]. A case report by Bressler et al. described a significant reduction in endometriosis-related refractory chronic pelvic pain when treated with 15 mg UPA for 3 months [105]. Contrary to the abovementioned case report, Donnez et al. described an excellent response to UPA treatment when administered over two 3-month courses in terms of fibroid reduction. On the other hand, there was no effect on ovarian endometrioma, with both conditions occurring simultaneously in the same patient [106]. Further studies in the form of well-designed RCTs are needed.

Vilaprisan (BAY 1002670) is a newer, potent, orally active SPRM. Its antagonistic effects are 5 and 10 times more potent compared with ulipristal and mifepristone, respectively [107]. Vilaprisan has been undergoing clinical trials for many indications, but the trial for endometriosis-related pain was prematurely closed. Even though it showed beneficial effects for treating uterine fibroids and heavy menstrual bleeding, its superior potency mentioned above makes this new SPRM unlikely to become a better option to treat endometriosis [108].

In summary, although some studies conducted with SPRMs in endometriosis have shown potential clinical use for symptom relief [109], no drug in this class is currently approved for clinical use in endometriosis therapy. Although mifepristone may have some benefits, the available evidence does not suggest the therapeutic value and clinical safety of other SPRMs for the long-term treatment of endometriosis [97].

### 3.9. Levonorgestrel-Intrauterine Device

The levonorgestrel-intrauterine device (LNG-IUD) was originally introduced for contraception purposes 50 years ago; however, LNG-IUD shows benefits in treating various benign gynecological conditions such as abnormal uterine bleeding (and subsequent iron-deficiency anemia), endometrial hyperplasia, endometriosis, leiomyomas, adenomyosis and coagulopathies [110,111,112]. Levonorgestrel (molecular formula—C_21_H_28_O_2_) is an estrane steroid derived from 19-nortestosterone and presents the second-generation progestin [110]. Apart from its contraceptive effects, LNG-IUD causes endometrial decidualization, thus reducing or suppressing menstrual flow [113]. Other secondary mechanisms that can have an effect on endometriosis-related pain are an overall decrease in prostaglandin production, mainly influenced by progestins, and downregulation of estrogen receptors in both glandular and stromal endometrial tissues [112,113].

The pharmacokinetical benefits of LNG-IUD were highlighted in the previously published guidelines by the European Society of Human Reproduction and Embryology (ESHRE), stating that clinicians may consider LNG-IUD as one of the options in alleviating endometriosis-related pain [114]. LNG-IUD has demonstrated a beneficial effect in increasing pain-free interval after surgery, compared to expectant management [115]. A recent prospective study by Zhu and colleagues demonstrated superior results in terms of recurrence prevention after endometrioma surgery with a combination that involved LNG-IUD and GnRH agonists [112]. Longitudinal studies found a significant reduction in the severity of dysmenorrhea after three years of follow-up [116]. However, irregular and intolerable bleeding associated with persistent pain during the first year of use can be a justified reason for treatment discontinuation and present possible disadvantages in clinical decisions [111]. Furthermore, this kind of treating endometriosis-related pain is still considered a second-line option and, in some countries, is considered an off-label therapeutic agent for this indication [114]. In conclusion, the LNG-IUD system can be suitable and should be considered in women who are dealing with strong hypoestrogenic adverse events from first-line agents used in symptomatic endometriosis.

### 3.10. Unconventional Therapy Options

Numerous research studies have demonstrated various beneficial effects of green tea on human health. The latest studies have shown that components of green tea, especially Epigallocatechin gallate (EGCG), act in preventing oxidative stress, reducing inflammation and angiogenesis, showing its potential to be used particularly in endometriosis treatment. By competitively binding to estrogen receptors in endometrial cells, EGCG inhibits the action of estrogen and prevents the growth of endometriotic lesions [117]. However, more thorough research and clinical trials involving endometriosis patients are required to confirm the benefits of green tea on the disease and its use as a possible therapeutic agent.

Curcumin, as an important anti-inflammatory agent, has been shown to have beneficial effects in patients with endometriosis. It can downregulate inflammation by reducing the activity of the NF-κB pathway in endometrial cells and inhibiting the expression of inflammatory factors, such as TNF-α, IL-1, IL-6, and IL-8. Moreover, it can have a direct impact on adhesion, apoptosis, and angiogenesis in endometrial lesions [118]. Its potential role in dietary prevention and disease management in women with endometriosis should be further investigated.

### 3.11. The Patients’ Pathway—From NSAIDs to Surgery

Optimizing medical therapy in patients with endometriosis is an important clinical challenge. Endometriosis treatment aims to achieve effective long-term outcomes regarding pain alleviation and recurrence rates and improve fertility and overall quality of life [119]. Given the complexity of its pathogenesis, a combination of interventions is often necessary for adequate treatment of this disease. Both hormonal and non-hormonal treatment options, which are previously discussed, are usually initially used for pain management and limiting the progression of endometriotic lesions. The use of NSAIDs to control inflammation and hormonal preparations to inhibit endometrial proliferation is still considered the main therapeutic option for this condition. Although the greatest NSAID dose tolerated by patients is often used for pain alleviation in the treatment of endometriosis, long-term use is not advised because of its potential adverse effects [17]. Regarding choosing between different options of hormonal therapy, an individualized approach and careful assessment of potential risks and benefits are needed for deciding on an adequate treatment course. Surgical removal of the endometrial lesions and adhesions is often beneficial and provides confirmation diagnosis by histopathology in women who do not respond to medical therapy or have severe symptoms [120]. Surgical evaluation and treatment are also considered in patients desiring pregnancy, although there is still no clear consensus about surgical treatment regarding infertility [119]. There is a lot of promising research conducted in the field of the development of new biomedical targets focusing on the cellular and molecular mechanisms causing endometriosis that could have profoundly beneficial effects on a patient’s diagnosis and treatment possibilities [121].

## 4. Materials and Methods

This review of the literature was provided in order to summarize data from relevant articles regarding the safety profile and clinical efficacy of endometriosis-related pelvic pain pharmacotherapy. Considering the nature of this research, an Institutional Review Board permission was not applicable. A literature search was conducted with the use of the PubMed and EMBASE electronic databases, focusing on identifying articles published in English between January 1990 and January 2023. 

Regarding the type of research, we included randomized clinical studies, observational studies, retrospective and prospective studies, and cross-sectional and case–control studies. We excluded from the analysis conference abstracts and case reports/case series. Furthermore, our included studies were ones that evaluate the drugs used or that are currently under investigation to treat endometriosis-related pelvic pain. Two reviewers (M.M. and M.Ć.) performed an independent search of sources using the following keywords: “endometriosis”, “pelvic pain”, and “chronic pelvic pain” alone or in combination with “medical treatment”, “medical therapy”, “progestins”, “levonorgestrel-intrauterine device”, “GnRH agonist/antagonist”, “ulipristal acetate”, “vilaprisan”, “safety profile”, and “pharmacokinetics”. The latest date of this search was on 1 February 2023. 

Titles and/or abstracts of studies retrieved using the search strategy, and those from additional sources, were screened independently by 2 review authors to identify studies that potentially meet the aims of this review. The full text of these potentially eligible articles was retrieved and independently assessed for eligibility by the other 2 review team members. Any disagreement between them over the eligibility of particular articles was resolved through discussion with a third (external) collaborator. Two authors independently extracted data from articles about study features and included populations, type of intervention (duration of therapy and drug posology), and outcomes. Any discrepancies were identified and resolved through discussion (with a third external collaborator where necessary). 

Due to the nature of the findings, we opted for a narrative synthesis of the results from selected articles.

## 5. Conclusions

Although there are substantial improvements in hormonal and non-hormonal therapy options, the majority of the currently available treatment options for endometriosis suppress ovarian function and do not represent a final solution for patients. Current strategies to manage endometriosis include pharmacological and surgical approaches and have to be tailored properly and timely with the primary goal of pain relief and restoring fertility. Adequate choice is made in accordance with the patient’s age, disease severity, and desire to preserve fertility. Based on the published evidence, clinicians should consider NSAIDs, COCs, and progestins as the first-line medical therapies. Compared with second-line options, such as GnRH agonists/antagonists or AIs, the abovementioned first-line options are well tolerated, efficacious, and exhibit lower overall price. Unfortunately, approximately 20% of patients report no improvement in pain with medical therapy, while discontinuation rates are affecting yet another 5–15% of patients because of significant, mostly hypoestrogenic adverse events. Considering these points, further research should be aimed at identifying novel pathways for targeted therapies, both hormonal and non-hormonal ones; in addition, available therapies should be tested for different routes of administration in order to evaluate whether they can cause fewer side effects. For instance, some pharmacological approaches such as GnRH agonists/antagonists or AIs, which are known to be associated with significant side effects, may be administered by vaginal approach, with a potentially reduced adverse effect rate due to a more stable steady state for drug release. In addition, the search for novel non-hormonal approaches should be aimed at addressing the control of endometriosis in women who desire pregnancy: indeed, hormonal treatments, both as a first-line approach or for prevention of recurrent disease after surgery, are contraceptives, and this represents the main drawback of this type of management. On this basis, another pivotal priority in the field should be the identification of new non-contraceptive and non-hormonal strategies to reduce as much as possible the symptoms and the progression of endometriosis, leaving the possibility of pregnancy.

## Figures and Tables

**Table 1:** Summary of the available medical options in the treatment of endometriosis-related pelvic pain, including their mechanism of action, adverse effects, and molecular structures.

Drug Category	Drugs	Mechanism of Action (in Endometriosis)	Adverse Effects/Toxicity	Molecule Structure of Representative Drug
**NSAIDs**	Ibuprofen, naproxen	Reversibly inhibits COX-1 and COX-2 → decreased prostaglandin formation	Gastrointestinal ulcers, edema, and renal impairment	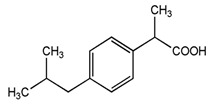
**Combined estrogen–progestin contraceptives**	Ethynil-estradiol combined with norethindrone, norgestrel, levonorgestrel, and desogestrel	Inhibits FSH and LH; decreases cell proliferation and enhances endometrial apoptosis	Nausea, vomiting, breast tenderness, weight gain, acne, depression, fatigue, breakthrough bleeding, thromboembolism, and increased risk of developing estrogen-dependent cancers	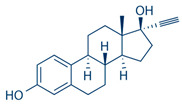
**Progestin-only preparations**	Norethindrone, medroxyprogesterone, and levonorgestrel	Inhibits FSH and LH and stimulates atrophy/regression of endometrial lesions	Weight gain, acne, breast tenderness, depression, hirsutism, nausea, and peripheral edema	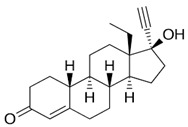
**GnRH agonists**	Leuprolide, goserelin	Inhibits steroidogenesis due to reduced FSH and LH levels	Hot flashes, vaginal atrophy, bone loss, and venous thromboembolism	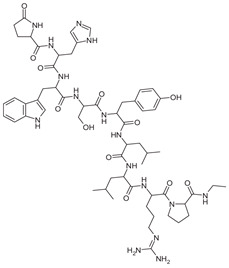
**GnRH antagonists**	Elagolix, relugolix, and linzagolix	Suppression of LH secretion, competitive action on GnRH receptors in endometrial cells → inhibition of growth and proliferation of endometrial tissue	Hot flashes, fatigue, weight gain, and decreased libido	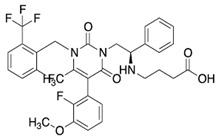
**SPRMs**	Ulipristal acetate, mifepristone, and asoprisnil	PR modulator-associated endometrial changes (PAEC) → endometrial antiproliferative effect	Nausea, vomiting, fatigue, diarrhea, headache, hot flushes, and loss of libido	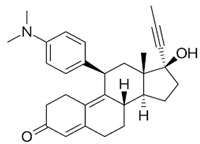
**Aromatase inhibitors**	Letrozole, anastrozole	Blocks conversion of androgens to estrogen → decreased endometrial proliferation	Arthralgia, myalgia, and decreased bone mineral density (BMD)	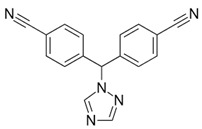

## Data Availability

Not applicable.
